# Shared decision-making in Cervical Cancer Care at Tikur Anbessa Specialized Hospital, Addis Ababa, Ethiopia: A mixed-methods study

**DOI:** 10.1371/journal.pone.0348172

**Published:** 2026-04-24

**Authors:** Sosina Workineh Tilahun, Adiam Nega, Lealem Wagaw, Adamu Addissie

**Affiliations:** 1 Department of Emergency, Critical, and Chronic Care Nursing, School of Nursing and Midwifery, College of Health Sciences, Addis Ababa University, Addis Ababa, Ethiopia; 2 Department of Health Systems Management and Health Policy, School of Public Health, College of Health Sciences, Addis Ababa University, Addis Ababa, Ethiopia; 3 Department of Epidemiology and Biostatistics, School of Public Health, College of Health Sciences, Addis Ababa University, Addis Ababa, Ethiopia; Population Council Ethiopia, ETHIOPIA

## Abstract

**Background:**

Shared decision-making is crucial for alignment of treatment options with patient values and preferences. However, currently in Ethiopia, shared decision-making in clinical care of cancer, in which cervical cancer is not exceptional, is not well understood.

**Aim:**

This study aimed to assess the perceived level of shared decision-making and its predictors in cervical cancer care at Tikur Anbessa Specialized Hospital in Addis Ababa, Ethiopia.

**Methods:**

We employed a convergent parallel mixed-methods study design from February 18 to May 23, 2025, at Tikur Anbessa Specialized Hospital. The study used interviewer-administered questionnaires for 203 cervical cancer patients and in-depth interviews for 15 cervical cancer patients and 10 clinical oncologists. Using SPSS v26, multiple linear regression analysis was used to determine significant predictors of the perceived level of shared decision-making, with statistical significance set at P < 0.05. Thematic analysis was conducted for the qualitative data.

**Results:**

The overall mean score for the perceived level of shared decision-making was 24.94 (± 9.12), with a range of 7–44, and the standardized mean score was 2.77 (± 1.01). The perceived level of shared decision-making had positive linear associations with increased trust in oncologists (0.32, 95% CI (0.21, 0.44); p < 0.001). Conversely, it had negative linear associations with rural residential area (−2.94, 95% CI (−5.34, −0.54); p = 0.02), unemployment status (−4.36, 95% CI (−6.96, −1.76); p = 0.001), and higher health literacy score (−6.89, 95% CI (−9.92–-3.86); p < 0.001). Thematic analysis revealed contextual, cultural, relational, institutional, and communicational factors influencing shared decision-making practice in cervical cancer care.

**Conclusions:**

The study emphasized the complex interplay of factors influencing the practice of shared decision-making in clinical care of cervical cancer. Therefore, understanding these dynamics may help to enhance the practice of shared decision-making in clinical cervical cancer care.

## Background

For decades, physicians were solely responsible for determining patients’ treatment plans, with little to no input from the patients [1,2]. However, contemporary medicine emphasizes that medical professionals are supposed to recommend clinical care interventions based on their expertise, which patients can accept or reject. This shift reflects a transformation in modern medicine and highlights the importance of involving patients in their clinical care, giving rise to the practice of shared decision-making (SDM) [2].

SDM suggests treatment decisions are made only after the patient fully understands the entire treatment plan, including its potential risks and benefits [3,4]. It empowers patients to make decisions based on their values, preferences, and experiences, solidifying its role as a fundamental aspect of patient-centered care [5–8]. Beyond its ethical and relational importance, implementing SDM in clinical practice has been associated with positive affective and cognitive outcomes [9]. Research from developed countries highlights its practical advantages, such as increased patient satisfaction, better cost-effectiveness, and a reduction in malpractice lawsuits [10,11]. Shared decision-making is not only a legal and ethical requirement strongly endorsed by medical associations but also a collaborative form of work that demands effort, negotiation, and institutional support to overcome power asymmetries and achieve true patient-centered care [12]. Conversely, inadequate application of SDM is associated with poorer health outcomes, lower quality of care, and increased healthcare utilization, which can be described as more hospital visits and resource use [13].

SDM is a crucial model in clinical decisions for serious illnesses, i.e., cancer care, where choices about interventions can directly impact treatment outcomes [4]. Delivering high-quality cancer care is important given the disease’s complexity, the impact of the treatment choice on patient’s quality of life (QoL), limited availability of evidence supporting clinical decisions, and patients’ priority regarding treatment [14]. Furthermore, SDM is often promoted as an ethical approach in cancer care due to the significant toxicity and life-changing nature of cancer therapies, followed by their psychological and practical impacts on both patients and caregivers [4,15].

Despite the growing availability of decision-making tools, many cancer patients find it challenging to make informed decisions about their treatment [4,5,16–18]. Empirical evidence identified several barriers to the effective implementation of SDM, including time constraints [19], the age of the patient [20], having no surgical intervention [21], low health literacy of the patient [19], provider uncertainty and attitude, the complexity of medical decisions, and the presence of multiple treatment options [14]. Additionally, a study from India revealed that cultural beliefs and prejudices influence the extent of participation and engagement of a patient in SDM practices [4]. However, growing attention to high-quality cancer care has increasingly emphasized the importance of SDM [4,6], where cervical cancer is not exceptional.

Cervical cancer accounts for over 85% of cancer cases and related deaths in low- and middle-income countries (LMICs). With an estimated 6,294 (13.6%) new cases and 4,884 (10.2%) deaths annually, cervical cancer is the second most frequent cancer type in Ethiopia, following breast cancer [22–24]. Having numerous forms of mild to severe physical, psychological, social, and spiritual impacts, and the consequences of its treatment modalities later in life, cervical cancer is also recognized as a life-threatening disease [20,25,26]. Consequently, SDM was witnessed to play a major role in determining the outcome of treatment modalities in the cervical cancer care by increasing levels of adherence to treatment intervention [27]. Hence, the foundation of the treatment is thought to be the woman’s and the clinician’s ability to communicate effectively [26].

Despite the leading stress of cervical cancer in Ethiopia, the perceived level of SDM and its determinants is limited in the literature. Therefore, this study aimed to assess the level of perceived SDM and its determinants in cervical cancer care at Tikur Anbessa Specialized Hospital (TASH), the country’s largest tertiary facility. The findings will provide evidence to strengthen patient-centered care, help healthcare providers identify barriers to SDM implementation, and inform quality-of-care indicators in clinical care of cervical cancer care. Additionally, the study will offer context-specific insights to the global literature on SDM in low- and middle-income countries and try to emphasize the importance of medical ethics in cervical cancer care.

## Methods

### Study setting, design and period

The study employed a convergent parallel mixed-methods design from February 18 to May 23, 2025, at the Adult Oncology Unit of Tikur Anbessa Specialized Hospital (TASH) to assess the perceived level of shared decision-making and its determining factors in the clinical care of cervical cancer.

TASH is one of the referral cancer centers in the country, and it was selected because it is a large referral center in the country for radiation therapy. The hospital’s status as a high-volume referral center for cervical cancer ensures access to a diverse patient population drawn from across the country, encompassing a wide range of sociodemographic backgrounds.

### Quantitative survey method

The sample size for the quantitative survey was calculated using the single population proportion formula. Due to the lack of prior studies on shared decision-making within similar socio-demographics, a 50% proportion, 95% confidence level, and 5% margin of error were assumed, consequently yielding 384. With a 10% non-response adjustment, it increased to 423. However, only 390 cervical cancer patients were on follow-up at TASH during the data collection period (per hospital records). Applying a finite population correction formula, the final sample size was 203. This meets the recommended threshold of 10–15 participants per predictor variable for adequate statistical power and avoids overfitting [28].

A total of 203 women with cervical cancer were approached consecutively; 4 provided incomplete responses, and 2 refused participation, resulting in 197 completed surveys. Inclusion criteria included participants who had undergone at least one round of adjuvant or neoadjuvant chemotherapy or radiation therapy during the data collection period, had no types of clinically diagnosed mental disorders, and were clinically stable. To enhance representativeness and reduce selection bias, data collection was strategically scheduled across various days and times. This approach minimized systematic exclusion by including patients with diverse treatment schedules, socioeconomic backgrounds, and geographic origins, thereby improving the generalizability of the study outcomes. Such a strategy is consistent with methodological recommendations for reducing selection bias in epidemiological studies [29].

Participants were provided with interviewer-administered questionnaires by two data collectors who have Master of Science degrees in clinical nursing and had one day of training by the principal investigator, and the Kobo Toolbox mobile application was used to collect the data. A pretest with 20 cervical cancer patients at St. Paul’s Hospital Millennium Medical College informed revisions, including simplifying technical terms, ensuring culturally appropriate language, removing redundant items, improving question order for logical flow, adjusting framing for respectfulness, and reducing survey length to minimize fatigue.

The questionnaires for the independent variables were adapted after numerous literature reviews constituting sociodemographic and clinical characteristics [14,16,19,20,30–33], the Trust in Oncologist Scale (TiOS) [34], and the Health Literacy Survey Questionnaire (HLS-EU-Q) [35].

TiOS measures four dimensions of trust, namely competency (Items 1, 6, 9, and 11), Honesty (Items 2, 3, and 12), Fidelity (Items 4, 5, 8, 10, and 15), Caring (Items 7, 13, 14, and 16), and Global (Items 17 and 18) on a 5-point Likert scale (1 = strongly disagree to 5 = strongly agree) [34]. Reverse scoring was applied to negatively keyed items 9, 11, and 13: ‘*Sometimes you worry that your doctor’s medical decisions are wrong,’ ‘Sometimes your doctor does not pay full attention to what you are trying to tell him/her,’* and *‘You have doubts whether your doctor really cares about you as a person*.’ Scores sum up higher scores, indicating stronger trust. The tool was documented to have Cronbach’s α = 0.9 in the Netherlands [36] and α = 0.92 at the University of Amsterdam [37].

Health literacy was measured by the European Health Literacy Consortium, which created the 47-item HLS-Q questionnaire [35]. It assesses three domains of health: health promotion (16 items), disease prevention (15 items), and healthcare (16 items). From very easy (4) to extremely difficult (1), a higher score implies a higher level of literacy. HLS-Q was found to have good internal consistency (α > 0.90) in a study across six countries, namely, Indonesia, Kazakhstan, Malaysia, Myanmar, Taiwan, and Vietnam; satisfactory item-scale convergent validity (item-scale correlation ≥ 0.40); and good construct validity [38], and it had good internal consistency in Albania (α > 0.92) [39].

The dependent variable was measured by shared decision making-9 (SDM-9). SDM-9 was developed in 2010 and was coined to measure patients’ perceived level of involvement in shared decision-making on a 6-point Likert scale from “completely disagree” (0) to “completely agree” (5), in which the range is from 0 to 45 points, indicating the higher the score, the higher the perceived level of shared decision-making. For internal consistency, the tool recorded (α > 0.92) in German [40] and (α > 0.92) in the United Kingdom [41].

The adaptation of the TiOS, HLS-Q, and SDM instruments into Amharic involved a multi-step process. First, the tools were chosen based on their previous validation in various oncology contexts and their alignment with the study objectives. Two independent experts, fluent in both English and Amharic and familiar with the medical terminologies, conducted the translations. These translations were then reviewed by a clinical oncologist and the principal investigators to ensure both conceptual and contextual equivalence. An expert, blinded to the original tools, performed a back-translation to the original language of English. Any discrepancies between the back-translated and original instruments were resolved through discussion [42]. The final Amharic versions of TiOS, HLS-Q, and SDM showed excellent internal consistency, with Cronbach’s alphas of 0.98, 0.98, and 0.97, respectively.

Data were exported into IBM SPSS Statistics version 26.0 (SPSS Inc., Armonk, New York), cleaned, and followed by statistical analysis. Missing data were assessed across all variables, and they were assumed to be missing completely at random based on visual inspection and lack of systematic patterns. To maintain analytical integrity, list-wise deletion was applied. And cases with missing values on any of the model variables were excluded from the analysis. To manage outliers, standardized residuals were inspected, and no case exceeded ±3 standard deviations.

In our descriptive analysis, we computed the averages and standard deviations for continuous variables, while for categorical variables, we reported frequencies and percentages. To investigate the relationship between the independent and dependent variables, we used linear regression. Linear regression was selected based on SDM’s score distribution and scale properties, which produce a continuous total score. Additionally, since the developers of the tool did not create a categorical classification, linear regression was further confirmed as the appropriate model to use.

We assessed the model’s assumptions. Histogram and Q-Q plot were used to evaluate residual normality, revealing symmetry and alignment with the diagonal, indicating a normal distribution. Furthermore, we performed Levene’s test for equality of variances indicated no significant differences across groups (Levene’s test, p = .210), supporting the assumption of homoscedasticity for our dependent variable. Although we performed formal tests like Kolmogorov–Smirnov and Shapiro–Wilk, they detected significant deviations in large samples which may contribute to the very sensitiveness of the tests. Thus, visual inspection was found to be crucial for practical interpretations of our results [43]. Additionally, with a sample size of n > 100, the Central Limit Theorem suggests that parametric analyses remain robust in the face of mild non-normality [44]. Cook’s Distance was calculated for all cases, ranging from 0.000 to 0.043, showing that no single observation had a significant impact on the model estimates. Given the sample size of 197 and absence of boundary estimates, Wald confidence intervals were considered appropriate for estimating parameter precision under the assumption of asymptotic normality.

### Qualitative interview methods

For the qualitative data, sample size determinations were guided by the concept of information power, the more relevant, specific, and rich the data are in relation to the study’s objectives, the fewer participants are required [45]. Consequently, 15 women with cervical cancer who had at least completed one round of neoadjuvant or adjuvant chemotherapy/radiotherapy and had not participated in a quantitative survey were recruited purposively for in-depth interviews (IDIs). And we purposively recruited 10 clinical oncologists who had been working with cancer patients for at least three years for key-informant interviews (KIIs). Saturation was monitored throughout the data collection, and it was determined to have been reached when no new themes or subthemes emerged after the 12th patient interview and the 8th clinical oncologist interview, with subsequent interviews confirming and reinforcing existing categories rather than introducing new concepts. This ensured that the dataset was both information-rich and adequately comprehensive to address the study objectives.

Two interviewers (i.e., the principal investigator, SWT, and a research assistant, AN) who have an educational background in health science and previous experience in conducting the qualitative study participated in the data collection. Data were collected in a separate designated room at the Outpatient Department (OPD) of the adult oncology unit. The IDIs lasted from 35:15–50:00 min, and the KIIs lasted from 55:23–1:15 hr.

To ensure trustworthiness of the study, several techniques were used. First, an unstructured interview guide was developed in relation to the research questions and pretested among cervical cancer patients and clinical oncologists at St. Paul’s Hospital Millennium Medical College. Second, the principal investigator, along with the research assistant, practiced how to pose and probe questions, listen, and record participants’ responses at the same time. Third, the principal investigator, along with the research assistant, spent enough time at the study setting communicating with cervical cancer patients and key informants. Fourth, adequate and appropriate time was taken during the interview (35:15 min-1:15 h) to discuss major thematic areas. Fifth, method triangulation (i.e., collecting data through two approaches—IDI and KII) and participant triangulation (collecting data from patients and clinical oncologists) were used to triangulate the data. Sixth, the context of the study setting and a diversity of participants, data collection processes, and analysis were thickly described.

Data analysis was conducted simultaneously with data collection. Data were debriefed on a daily basis with the research assistant to ensure data saturation, completeness, and consistency with the simultaneous incorporation of data from field notes. Interviews were audiotaped, and verbatim transcription was conducted by the principal investigator and research assistant (i.e., data were collected in Amharic). The transcribed data were translated to English, and the translated data were cross-checked for completeness and consistency in meaning with the transcriptions.

Then reading and rereading of the transcription were conducted to extract important concepts related to the research objective and the best quotes. The ATLAS.ti 9.1 software package was used to perform line-by-line coding. To enhance the credibility of the analysis, the principal investigators and research assistant independently double-coded the transcripts. Any discrepancies in coding were thoroughly discussed and resolved through consensus, leading to adjustments in the codebook for improved clarity and consistency. Subsequently, the entire dataset was coded using the revised codebook and analyzed through an inductive thematic approach [46]. Finally, the findings were presented with themes, sub-themes, and best quotations derived from the data.

### Data integration and synthesis method

After the completion of the independent data analyses of both quantitative and qualitative data, the generated primary inferences were merged using the iterative joint display [47]. This stage allowed us drawing meta-inference from table of findings generated from the two analysis [48]. This approach allowed us to integrate statistical associations with thematic insights, facilitating complex and detailed interpretation of how shared decision-making is shaped within cervical cancer care. This systemic method of analysis also enabled identifying findings that complement and contradict each other. Further it enabled us to introduce qualitative determinants of shared decision making in clinical care of cervical cancer

### Ethical considerations

This study was conducted according to the guidelines of the Declaration of Helsinki. Ethical clearance was granted by the Research Ethics Committee (REC) of the Department of Epidemiology and Biostatistics at the School of Public Health, College of Health Sciences, Addis Ababa University (Approval Protocol Number: REC-EB-SPH-001/2025), approved on January 22, 2025. Capacity to consent was assessed by trained data collectors, who provided participants with written information about the study prior to participation. They assessed participants’ understanding of the study’s objectives, procedures, risks, and the voluntary nature of participation. Participants were given the opportunity to ask questions, and consent was obtained and documented only after ensuring informed and voluntary consent.

Written individual informed consents were obtained from each participant for the quantitative survey. For those unable to read or write, consent was obtained from a witness. Additionally, verbal consents for the qualitative interviews were audio recorded alongside the written informed consent. Confidentiality was maintained by omitting any identifying information, such as participants’ names from the dataset. Audio recordings were stored on the principal investigator’s password-protected personal computer, while transcripts were securely kept on encrypted drives accessible only to the research team. Anonymity was preserved during analysis, and quotations were selected to prevent deductive disclosure. All methods adhered to relevant guidelines and regulations.

## Results

### Result from the quantitative data

#### Socio-demographic Characteristics of Participants.

The overall response rate of the survey was 97% (197/203 respondents), aged in the range of 31–80 (54.44 ± 10.41). The majority of the respondents had not pursued formal education and were unemployed, 109 (55.3%) and 148 (75.1%), respectively. Nearly equal number of respondents were married and lived in urban areas, 126 (63.9%) and 124 (62.9%). Nearly 90% of the respondents received the service through health insurance, 176 (89.3%). (Table 1)

**Table 1:** Socio-demographic characteristics of women with cervical cancer at TASH, Addis Ababa, Ethiopia (n = 197).

Socio-demographic Characteristics	Frequency	Percentage
**Age (years)**		
<50 years old	71	36.00
≥50 years old	126	64.00
Mean (±SD)	(54.44 ± 10.41 years old)	
**Area of Residence**		
Urban	124	62.90
Rural	73	37.10
**Religion Affiliation**		
Orthodox	143	72.60
Protestant	21	10.70
Muslim	32	16.20
Other*	1	0.50
**Educational status**		
Did not join formal education	109	55.30
<High school	56	28.40
≥High school	32	16.30
**Occupation**		
Unemployed	148	75.10
Employed	49	24.90
**Average Monthly Income in ETB** Mean (±SD)	(3014.96 ± 2489.07ETB)	
**Marital status**		
Never been married (Single)	14	7.20
Married	126	63.90
Separated	57	28.90
**Source of treatment expense**		
Health insurance	176	89.30
Self-sponsored	21	10.70

Footnote: *Jehovah Witness; ETB: Ethiopian Birr; Rural: those who are living out of Addis Ababa city; Urban residents: those living in Addis Ababa

#### Clinical characteristics of participants.

The majority of the participants were diagnosed with stage III and had underwent surgery, 92 (46.7%), and 128 (65%). More than half of the respondents received both chemotherapy and radiation, and reported ECOG (Eastern Cooperation Oncology Group) status of II, 114 (57.9%) and 122 (61.9). On average, the participants had 16.27 (±10.81) consultation rounds, and it had been 34 months (±38.12) since their diagnosis (Table 2).

**Table 2:** Clinical characteristics of women with cervical cancer on adjuvant therapy at TASH, Addis Ababa, Ethiopia, (n = 197).

Clinical Characteristics	Frequency	Percentage
**Stage of the disease**		
Stage I	6	3.00
Stage II	53	26.90
Stage III	92	46.70
Stage IV	46	23.40
**Surgical Intervention**		
Yes	128	65.00
No	69	35.00
**Time Since Diagnosis in months** Mean (±SD)	34.25(±38.12)	
**ECOG**		
I	43	21.80
II	122	61.90
III	28	14.20
IV	4	2.00
**Type of treatment**		
Chemotherapy	36	18.30
Radiotherapy	44	22.30
Chemotherapy and Radiotherapy	114	57.90
Other*	3	1.50
**Round of Consultations** Mean (±SD)	16. 27 (±10.88)	

Footnote: ECOG: Eastern Cooperation Oncology Group; * Radiation and Surgery

#### Descriptive statistics for trust in oncologist and health literacy score.

The overall mean level of trust in oncologists (TiOS) in this study was 69.95 (±9.41), with a scale range of 34–90 and a standardized mean score of 3.89 (±0.52). The average health literacy score for the women in our study was 114.37 (±25.98), with a minimum score of 75 and a maximum score of 188. The standardized mean of the health literacy score was 2.43 (±0.53). The health literacy score was categorized as low, medium, and high health literacy score based in the percentile groupings. And the majority of participants had a low health literacy score, 68 (34.50). Table 3

**Table 3:** Descriptive statistics for Trust in Oncologist Scale and Health Literacy Score.

Measures	Mean (SD)	Range (Max-Min)	Standardized Mean (SD)	Category	Frequency (%)
Trust in Oncologist Scale	69.95 (±9.41)	34-90	3.89 (±0.52)	–	–
Health Literacy Score	114.37 (±25.98)	75-188	2.43 (±0.53)	Low Health Literacy Score	68 (34.50)
				Medium Health Literacy Score	63 (32.00)
				High Health Literacy Score	68 (33.50)

#### Level of shared decision making.

The SDM scale was initially coded from “completely disagree” (1) to “completely agree” (6). Thus, the items were recoded back to the original scale, resulting in an average score of 24.94 (SD = 9.12), with a range of 7–44 and a standardized mean score of 2.77. One item scored above 3.00: “***My doctor made it clear that a decision needs to be made***,” with a mean of 3.01 on a scale of 1–5, while the lowest score was for “***My doctor and I selected a treatment option together,***” with a mean score of 2.59 on a scale of 0–5. (Table 4)

**Table 4:** Descriptive statistics of perceived level of shared decision making among women with cervical cancer at TASH, Addis Ababa Ethiopia, (n = 197).

Descriptive Statistics				
**Variables**	**Minimum**	**Maximum**	**Mean**	**Std. Deviation**
My doctor made it clear that a decision needs to be made	1	5	3.01	1.12
My doctor wanted to know exactly how I wanted to be involved in making the decision	1	5	2.84	1.17
My doctor told me that there are different options for treating my medical condition	1	5	2.94	1.14
My doctor precisely explained the advantages and disadvantages of the treatment options	0	5	2.59	1.19
My doctor helped me understand all the information	0	5	2.83	1.13
My doctor asked me which treatment option I prefer	0	5	2.68	1.10
My doctor and I thoroughly weighed the different treatment options	0	4	2.68	1.07
My doctor and I selected a treatment option together	0	5	2.60	1.10
My doctor and I reached an agreement on how to proceed	0	5	2.77	1.05
Total mean score of SDM	24.94 (±9.12)			

#### Predictors of shared decision making.

We conducted a simple linear regression analysis to identify the relationship between each independent variable and the perceived level of shared decision-making practice, aiming to identify variables for inclusion in multiple linear regression. Consequently, age distribution, area of residence, educational level, occupational status, average monthly income in Ethiopian birr, average time since diagnosis in months, Health Literacy Score Distribution, and trust in oncologists (TiOS) had P < 0.25 in simple linear regression analysis. Thus, eight variables were included in the multiple linear regression. The model accounted for 46.0% of the variation in shared decision-making, while the remaining factors explained 54.0% (R-squared = 0.460, adjusted R-squared = 0.431). The overall model was statistically significant (F (10,186) = 15.876, p < .001), indicating that the predictors collectively provide meaningful explanatory power.

When controlling for other variables in the model, women residing in rural areas scored nearly 3 units lower on perceived shared decision-making compared to those in urban areas (−2.94, 95% CI (−5.34, −0.54); P = .017). Similarly, unemployed women scored nearly 4.36 units lower than employed women (−4.36, 95% CI (−6.96, −1.76); P = .001). Women with high health literacy reported almost 7 units lower perceived shared decision-making compared to those with low health literacy (−6.89, 95% CI (−9.92, −3.86), P < .001). In contrast, each one-unit increase in trust in oncologists was associated with a 0.32‑unit increase in shared decision-making (0.32, 95% CI (0.21, 0.44), P < .001). Furthermore, all items on the SDM‑9 were significantly and negatively associated with mean health literacy scores, indicating a consistent inverse relationship across the scale. (Table 5)

**Table 5:** Multiple linear regression analysis predicting perceived level of shared decision in cervical cancer care at TASH, Addis Ababa, Ethiopia,(n = 197).

Variables	Unadjusted Unstandardized ß 95% CI of β	Adjusted unstandardized ß 95% CI of β	P-Value
**Age Distribution**			
≥50 years old	2.16 (−0.50, 4.82)	2.11 (−0.02, 4.24)	
<50 years old	**Ref**	**Ref**	
**Area of Residence**			
Urban	**Ref**	**Ref**	
Rural	−5.77 (−8.30, −3.23)	−2.94 (−5.34, −0.54)**	**0.017**
**Educational level**			
Didn’t join formal Education	**Ref**	**Ref**	
<High school	5.07 (2.28, 7.87)	2.22 (−0.35, 4.78)	
≥High School	7.61 (4.20, 11.03)	0.25 (−3.30, 3.81)	
**Occupational Status**			
Employed	**Ref**	**Ref**	
Unemployed	−4.45 (−7.35,-1.54)	− 4.36 (−6.96, −1.76) **	**0.001**
**Average Monthly Income per 1000 ETB**	1.00 (0.00,1.00)	−0.000 (0.001, 0.000)	
**Time since Diagnosis in months**	0.03 (−0.01, 0.26)	−0.02 (−0.04, 0.01)	
TiOS* (69.9 (±9.43)	0.48 (0.36,0.60)	0.32 (0.21, 0.44)**	**0.00**
**Health literacy Score**	−0.24 (−0.28,-0.20)	−0.18 (−0.23, −0.13)**	
Low Health Literacy	**Ref**	**Ref**	
Medium Health Literacy	−2.89 (−5.53, −0.25)	−0.92 (−3.54, 1.71)	
High Health Literacy	−11.72 (14.32,-9.11)	−6.89 (−9.92, −3.86)	**0.00**

Footnote: TiOS: Trust in Oncologist Scale; ETB: Ethiopian Birr Ref: Reference. The dependent: Standardized mean score of SDM Scale. Model statistics: R = .679, R² = .460, adjusted R² = .431, and standard error of the estimate = 6.88. **Statistical significance of p < .05. Adjusted Models: average monthly income (ETB), time since diagnosis (months), area of residence (rural), occupation (unemployed), educational status (≤ high school, > high school), age group (≥ 50 years old), health literacy score distribution (Low health literacy Score, Medium Health Literacy Score, and High Health Literacy Score), and mean trust in oncologists. Multicollinearity test: maximum VIF = 1.84; all VIFs < 10). Normality test: Q-Q plots, Histogram, and Levene’s test, p = .210.

### Result from the qualitative data

For the qualitative data, the age range of the participating women was 39–56 years old, with an average age of 47.6. Key informants ranged in age from 32 to 44 years old, with an average age of 32.8 years old. None of the key informants had received training regarding clinical decision-making or communication. Six of the ten clinical oncologists, or 60% of the participants, were men.

Qualitative data were thematically categorized into barriers and facilitators of shared decision-making practice in cervical cancer care. Subthemes included contextual, cultural, relational, institutional, and communicational barriers, along with contextual and relational facilitators.

### Theme I: Barriers to shared decision making

#### Sub-theme I: Contextual Barriers.

The financial status of the women was reported to be linked to the shared decision-making practice. The illness has made many women lose their livelihood, leaving them financially constrained. Consequently, affordability, not comprehension, drives treatment choices, marking a troubling disconnect between informed consent and economic survival.

“*Sometimes, the decision of the patients is determined by their financial ability rather than the information they have. And here, a lot of women have already stopped their regular jobs due to the disease. So, they listen to your suggestions just because they can afford it; it is not because they understand it.*” (**P1, Clinical Oncologist, Age 40**)

Additionally, with the intention of protecting the patient from emotional harm, physicians may not fully discuss expensive treatment options. And this comprises autonomy and informed consent, the key pillars of SDM, further leading to the ethical complexity of selective disclosure.

“*Shared decision-making is much more difficult for radiotherapy than for chemotherapy. In this hospital, radiotherapy is also available in the private wing, but it is expensive. So, we mostly don’t tell them about the option of a private wing if we believe they cannot afford it. Because telling them would just create frustration!* *But, to your surprise, most of the time they already know, and that is another frustration.*” (**P4, Clinical Oncologist, 34**)

The intersecting vulnerabilities, older age and physical frailty, were explained to influence the practice of shared decision-making. Because these intersections compromise their ability to comprehend the situation.

“*Sometimes the patients may be old ages so that they don’t understand us that much and may also be very weak, so they don’t understand the process.*” (**P3, Clinical Oncologist, Age 32**)

#### Sub-theme II: Cultural barriers.

Cultural norms shape the patient-doctor dynamic, particularly in the tradition of “*doctors know best.*” This internalized sense of hierarchy suppresses shared decision-making practice, making patients suppress their concern and feel less empowered in their treatment decision.

“*I think our society and the way we grow up prevent us from fully communicating with our physicians. Because we grew up thinking physicians know better for us than we do for ourselves.*”**(P14**, **Patient, Age 42**)

Similarly, cultural expectations of the patient may influence the patient’s willingness to engage in shared decision-making practice. Patients might assume expert guidance from their doctors as a sign of uncertainty by equating professional ethics with professional inadequacy.

*“Now I encounter a patient who was given options to select from chemotherapy and radiation therapy, and she was very offended. She thought the doctor was incompetent to tell her the best management. So, in these kinds of situations, it will be difficult to make patients participate in the process of shared decision-making.”* **(P7, Clinical Oncologist, Age 37)**

#### Sub-theme III: Relational barriers.

Patients have witnessed that when their doctor is not involved and attentive during patient-doctor communication, it leads to irritation. Such an experience can leave patients feeling unseen, unheard, and devalued, undermining trust and the therapeutic relationship. Consequently, it reflects a deep expectation that medical care needs to be rational and patient-centered.

“*There was a young physician in the room when I entered. And he began speaking to me while writing on a piece of paper in front of him. Finally, he looked up and gave me the paper and told me to go to the pharmacy. Then I became offended and told him that he does not fit into the profession.*” (**P6, Patient, Age 42**).

Less kind treatment from their health care provider may make patients internalize a sense of unworthiness and fear of being misunderstood. And it does not only compromise the quality of shared decision-making in the clinical practice but also make the patients withdraw from any form of medical communication.

“*Some physicians don’t talk to you respectfully. They also don’t treat you properly. When they don’t talk to you properly, you even let go of what you think of asking them in the consultation room.”* ***(P9, Patient, Age 48)***

#### Sub-theme IV: Institutional Barriers.

Tertiary hospitals in Ethiopia, which are serving as cancer centers, are also teaching hospitals at the same time. Thus, for the sake of active teaching purposes, patients’ privacy and confidentiality may come under question. Yet, it may make the women lose control over their body, suppressing appropriate practice of shared decision-making along the way.

“*Let me tell you, I have reservations about the examination process. They made me open my cervix, and there were a lot of students, and they teach the students about the cervix like that. So, they made me sleep on the couch without clothes on, opening my leg for over 30 minutes. So, do you think I will want further communication with that physician? No. I will never.*” **(P11, Patient, Age 56)**

Similarly, patients may be compelled to meet different kinds of physicians in every follow-up. This practice makes them get inconsistent information about their illness. It further increases the burden on the patients by making them share their history in every follow-up, which is boring for them and compromise their trust in the health care system.

*“You always meet a new doctor when you come for a follow-up. So, you need to talk all over again with the new doctor. Communicating with each of them all over again is the boring part of the follow-up.*” **(P7, Patient, Age 49)**

Similarly, the patient overflow and time constraints may make the patients fail to ask questions and express their concerns and preferences, which are bases for the provision of subjective care and building shared decision-making practice.

*“Additionally, given the high patient load, they may not have adequate time for extended discussions with each patient.”* **(P8, Patient, Age 54)**

#### Sub-theme V: Communicational Barriers.

Health care providers have noticed ethical and relational tension that rises when family caregivers/attendants try to control patient-doctor communication. Although this protective instinct stems from a sense of care and concern, it limits the patient’s ability to fully understand their condition, which further compromises the relationship between the patient and the health care provider.

“*Sometimes, the attendants think that telling the patient everything about the disease may hurt the patient’s emotions. So, they ask you not to tell the patients about their diagnosis. They will even tell you that they will discuss it with other caregivers back home. And most of the time this makes it difficult for us to communicate with patients freely*.” **(P5, Clinical Oncologist, Age 36)**

When physicians use clinical terms or language, patients can be reluctant to participate in open discussion with the provider, as it causes them to feel less adequate. And this can make the patients bear increased emotional weight and uncertainty.

“*I asked why I got swollen. I have already had surgery. Why did I have the swelling? Then he started explaining, but I didn’t listen to him attentively since it was mostly clinical language****.”* (P2, Patient, Age 50)**

Limited communication can lead patients to have a poor knowledge about their disease and its specific outcomes. This can force the patients to seek peer support, likely shaped by anecdotal experiences and emotional narratives rather than balanced medical guidance. Additionally, turning to non-medical information sources can exacerbate their condition due to the overwhelming nature of information overload.

“*I wanted to ask the physician about my disease, but the physician didn’t give me much of an answer. He just told me that I need to have radiation. Then when everything became complicated for me, I started to visit women who were already diagnosed and were receiving radiation to ask for information about radiation. And visiting and talking to them made me fear the radiation.”* **(P10, Patient, Age 45)**

While shared decision-making emphasizes informed engagement, some patients become passive in their engagement. And this shift away from the medical decision is from the preferences of the patients.

“*The patients may not want to know about the disease. Sometimes, the patients may not want to know the status and the stage of the disease. Some may be ‘decide by yourself’ type of patient***.**” **(P10, Clinical Oncologist, Age 35).**

When there is one-way information flow, like in passive care practice, the patient may fail to feel the need to participate in communication with the physicians. Further, in this directive way of communicating, where physicians focus solely on instructing or directing the patient, they will miss the opportunity to collaborate with the patient and build trust. It may also lead patients to feel like their preferences, thoughts, and concerns are disregarded, making them more frustrated and uncertain.

*“Because no one asks you what you feel? They just say, ‘Take this,’ ‘Do this.’ Now I fear, what if the disease comes back tomorrow? I always fear that. But I don’t ask the doctor. I have never asked the doctor, as they don’t tell me clear and true things about my disease.”* **(P5, Patient, Age 52)**

### Theme II: Facilitators of Shared Decision Making

#### Sub-theme I: Contextual facilitators.

Health care providers noted a clear distinction in the engagement level of patients in shared decision-making, depending on their residential area. People from urban areas have differences in communication style and increased engagement in shared decision-making. Further, those populations come prepared with questions and a willingness to communicate, as they may have increased sources of information.

“*Now when our patients come from urban areas, they ask you a lot, and it’s also easy for us to communicate with them.*” **(P7**, **Clinical Oncologist, Age 37**).

Patients noticed that a solid understanding of their cases can empower them to participate in the shared decision-making process. Further, this increased comprehension can enhance their confidence in their interaction with their healthcare providers.

**“***Now there was a person who told me not to go to treatment, rather to go to holy water. Then I told them I would go to both holy water and treatment. So, knowing about the disease helps you a lot in knowing what to do next and understanding your doctor.*” **(P15, Patient, Age 39)**

*“Lack of awareness about the disease and medications also prevents us from asking for more information.*” **(P3, Patient, Age 45)**

Similarly, providers may tend to support the preferences of the more knowledgeable patient because, as the knowledge makes them empowered, they will be more involved in expressing their preferences and concerns, which can simplify the patient and health provider communication.

“*There are some patients who ask intensively. Those who come after searching for some information and are educated ask you in detail.*” **(P8, Clinical Oncologist, Age 32)**

#### Sub-theme II: Relational Facilitators.

Empathetic care makes the patients feel valued and respected, and it will create an environment in which the patient can discuss freely with their physician, further making the patient feel more supported and cared for. Further, reassurance can decrease patients fear and encourage them to further communicate with their physician. Because it gives patients space and time to reflect on their values and concerns.

“*He assured me that if we detect it early, there is no need to fear, as timely treatment allows people to live with it. He told me, ‘Mother, this is not something you need to be afraid of.’ After that, I began asking him unnecessary questions, even bringing up things I heard from other patients.****”*** **(P11, Patient, Age 56)**

Further, the compassionate care approach of the providers helps them build trust with their patients. Because patients who trust their providers tend to share information with their providers, creating a more conducive environment for the treatment process and making patients more likely to follow treatment recommendations and adhere to care plans.

“*Building trust with your patients will solve a lot of problems related to communication. Because building trust will increase the patient’s adherence to the medications and make the patient feel at ease around you.*” **(P6, Clinical Oncologist, Age 38).**

Emotional stability of the patient has also been witnessed to contribute to the increased practice of shared decision-making. Because the patient’s ability to manage anxiety can help in facilitating constructive conversation about the potential path forward. Further, it can enable patients to involve themselves in meaningful discussions with their physicians.

“*Since I am emotionally good, I was not very anxious with my physician about the futurity of the disease. Because the years you live are given by God. If he wants to, he will cure me.*” **(P4, Patient, Age 47**)

### Integrated survey results and qualitative findings

Table 6 shows a side-by-side joint display of the quantitative and qualitative analyses, revealing a comprehensive understanding of the data. The merging of the primary inferences enabled us to appreciate how SDM practice is being influenced in clinical care of cervical cancer.

**Table 6:** Fourteen meta-inferences resulted from the iterative joint display analysis process: ‘family involvement,’ ‘patient-doctor communication style,’ ‘limited communication of physicians,’ ‘preferences of patients for passivity,’ ‘systematic constraints,’ ‘financial vulnerability,’ ‘selective disclosure,’ ‘comprehension difficulties,’ ‘cultural norms (hierarchies and expectation),’ ‘effect of emotion on SDM,’ ‘effect of residential area on SDM,’ ‘knowledge about the disease,’ ‘effects of physician approach on SDM,’ and ‘effect of trust on SDM.’.

Quantitative data	Meta-inference	Qualitative data
The overall mean score for perceived shared decision-making was 24.94 (± 9.12), with a range of 7–44 and a standardized mean of 2.77 (± 1.01), showing moderate patient participation.	**Family involvement** (confirmation)	The participating health care providers explained the culturally embedded relational tensions in which a family caregiver tries to control patient-doctor communication, reducing direct participation of patients in their care process.
	**Patient-doctor communication style** (confirmation)	Patients seemed to be less engaged in clinical decisions when the physicians used technical terms (not a patient-centered approach) and became more directive than involving.
	**Limited communication of the physicians (**expansion)	Patients seemed to be forced to look for non-medical advice due to limited communication from their physicians. As a result, they experience information overload, which in turn will increase their fear.
	**Preferences of patients for passivity** (expansion)	Physicians observed that some patients prefer to deliberately avoid information about their disease, in which they defer decisions entirely to the physicians.
	**Systematic Constraints** (confirmation and expansion)	Patients are well aware of patient overload, compromised privacy, and inconsistent communication in a teaching hospital, which make their clinical engagement less.
Statistically, unemployed patients reported a significantly lower perceived level of shared decision-making. (β = −4.36; P = 0.001)	**Financial vulnerability** (Confirmation)	The health care providers explained how financial vulnerability gets induced by loss of job, profoundly shaping treatment choices, as affordability rather than comprehension may drive clinical decision-making practice.
	**Selective disclosure** (expansion)	The participating oncologists further acknowledged that the financial status of the patients determines the extent of information they provide to the patient.
There is no statistical association between age and SDM, nor between ECOG and SDM.	**Comprehension difficulties** (refinement)	The healthcare providers described how old age and weak physical status of the patient limit the practice of SDM in cervical cancer care by compromising the patient’s comprehension and understanding.
–	**Cultural norms (hierarchy and expectation)** (Introduction)	The patients and physicians described how cultural norms and approaches shape the patient-doctor dynamic through the tradition of ‘*doctor knows best*.’ The participating physicians explained that doctors are expected to be perfect, and offering choices to the patients might be considered professional inadequacy by the patient rather than professional ethics.
–	**Effects of emotion on SDM** (Introduction)	Patients seemed to be aware of how emotional stability reduces anxiety and fosters their communication with their physician.
Women from rural areas were significantly less engaged in shared decision-making compared to their urban counterparts (β = −2.94; P = 0.02).	**Effect of residential area on SDM** (confirmation)	The healthcare providers highlighted that patients from urban areas arrive with questions that draw from diverse sources of information and have a willingness to engage in communication with the providers.
Patients with higher health literacy scores reported significantly lower participation in SDM practice. (β = −6.89; P < 0.001)	**Knowledge about the disease** (contraindication)	Both patients and health care providers highlighted that knowledge and awareness about the disease increase practice of SDM in clinical care.
Higher trust levels of patients were significantly associated with higher patient involvement in SDM practice (β = 0.32; P < 0.001).	**Effects of physician approach on SDM** (Confirmation)	The women reported how inattentive or dismissive physician behavior has affected their trust in the clinical care. The women highlighted empathetic and reassuring communication from the physician to create more space to build trust and foster the practice of SDM.
	**Effect of trust on the SDM** (Expansion)	The participating healthcare providers explained the contribution of trust in the facilitation of treatment adherence and ease of communication.

First, the qualitative analysis confirmed that SDM in clinical care of cervical cancer is shaped by multiple communication barriers and cultural dynamics, which was shown in our quantitative analysis to be moderate. We confirmed how family involvement often creates relational tensions, with caregivers attempting to control patient–doctor communication and thereby limiting patients’ direct participation. Patient–doctor communication styles also played a role, as physicians’ reliance on technical language and directive approaches reduced patient involvement in their care process. This findings expanded our understanding of how limited physician communication further forced patients to seek non-medical advice, which led the patients to information overload and heightened fear. Expanding on this, we understood that some patients’ deliberate preference for passivity, avoiding disease-related information and deferring decisions entirely to their doctors, further limited the SDM in clinical care of cervical cancer. Systemic constraints within teaching hospitals such as patient overload, compromised privacy, and inconsistent communication due to physicians’ rotation were also confirmed and expanded upon, showing how institutional structures diminish opportunities for meaningful patient involvement in SDM.

Second, statically unemployed patients reported a significantly lower perceived level of shared decision-making compared to their employed fellow patients. Similarly, qualitative analysis confirmed financial vulnerability, often induced by job loss, profoundly shapes treatment choices, as affordability rather than comprehension may drive clinical decision-making. This expands our understanding that patients’ financial status directly influences the extent of information disclosed, with selective communication practices emerging as physicians tailor the amount of detail they provide based on the perceived financial status of the patient.

Third, although there was no statistical association between age or ECOG performance status and patients’ perceived level of shared decision-making (SDM), the qualitative finding refined our understanding by noting that comprehension difficulties remain a practical barrier as health care providers described how older age and weakened physical status compromise patients’ understanding and engagement in cervical cancer care decision-making.

Fourth, the qualitative findings newly introduced the role of cultural hierarchies, expectations, and emotional stability in shaping shared decision-making (SDM). Thus, we came to understand how cultural norms, which put physicians as unquestioned authorities in clinical encounters and demand doctors to appear flawless, shape the practice of SDM in clinical care of cervical cancer. Furthermore, this finding sheds light on how emotional well-being functions as an enabler of SDM practice in clinical care of cervical cancer.

Fifth, in the current study, patients from rural areas had a significantly lower perceived level of decision-making when compared to patients from urban areas. In our qualitative analysis we confirmed that urban patients are benefiting from greater information access and confidence in dialogue, which makes them get engaged in clinical decision-making more.

Sixth, the statistical result on the health literacy score appeared to contradict our qualitative findings, which emphasized that knowledge and awareness about the disease generally increase SDM in the clinical care of cervical cancer. This tension made us understand that while greater health literacy is expected to foster engagement, in practice it may lead to more critical evaluation of physician communication or heightened expectations, which paradoxically reduce patients’ perception of their involvement in SDM.

Seventh, in the current study, higher trust levels of patients were significantly associated with higher patient involvement in their care process. Similarly, our qualitative findings confirmed how inattentive physician behavior undermines trust in clinical care and empathetic and reassuring communication fosters it. Expanding on this, we came to understand that trust in clinical care facilitates treatment adherence and eases patient-doctor communication.

## Discussion

Using a convergent mixed-methods design, the current study found a perceived level of SDM of 24.94 (±9.12). While SDM scores have been reported in other countries [49,50], direct numerical comparisons are limited due to differences in cancer types, healthcare systems, and measurement contexts. Therefore, our discussion emphasizes the patterns and predictors observed within the present setting.

Consequently, our qualitative data revealed the family caregiver role as both supportive and controlling, which might dilute patient-doctor interaction. This dual role of family caregivers might decrease the practice of SDM in the clinical care of cervical cancer. Additionally, a couple of studies revealed how a family-centered care approach happened to be culturally embedded but limited patient autonomy [51,52]. Furthermore, a qualitative study from Ethiopia showed how family and relatives intermediated clinical consultation, both facilitating and constraining patients’ direct participation in their clinical care [52]. A similar concept has been observed in global oncology, as families are key actors in the clinical care of cancer patients, which is found to overshadow patient preferences [53].

Moreover, patient-provider communication in the Ethiopian health care setting, which has been reinforced by our qualitative findings as directive and relying on clinical jargon, has also been indicated to contribute to the moderate practice of SDM in the clinical care of cervical cancer. Because directive communication in clinical care might be perceived by patients as authoritative rather than collaborative, which might make the patients vulnerable to misinformation [51]. Thus, expanding on this, we understood patients’ deliberate preference for passivity, which further limited their participation in their clinical care. Such passive coping strategies have been documented in other oncology settings where patients assume top-down decision-making as sufficient, further ensuring power asymmetries [53].

Additionally, our qualitative findings have informed how systematic constraints limit patient engagement in their clinical care. Similarly, a study in Ethiopia shows how an overcrowded oncology clinic, time constraints, and teaching hospital environment further exacerbated the issues, as prioritizing would be given to efficiency and clinical teaching over patient-doctor individualized communication [54], which might push them to look for other non-medical information through their informal networks.

Women who were not actively employed had significantly lower SDM practice, which was demonstrated in our thematic analysis as financial constraints of the patients affecting their SDM practice. This shed light on the contributions of the socio-economic disparities to health care access and quality [8]. Furthermore, a study read that the unemployment status of patients results in their diminished confidence in negotiating with healthcare providers [55]. As a result, those patients may not voice their preferences and questions about clinical recommendations, feeling they are not entitled to ask. Thus, the occupational status of patients is one of the social determinants of SDM practice, which reminds us of the necessity of a context-specific SDM model, which enables disadvantaged patients to participate in their clinical care reasonably. Although financial toxicity hinders treatment adherence and increases early mortality [56], modern oncology has introduced expensive treatments. Nevertheless, caregivers might be involved in selective disclosure, focusing on affordable treatments to protect patients from distress and still ensuring an informed consent process [57].

Although SDM had no statistically significant associations with ECOG and age, our qualitative data refined our understanding by noting how comprehension difficulties contribute to limited participation in the care process. Consequently, clinicians described older patients as often cognitively fatigued, physically weak, or less able to comprehend complex medical information. Furthermore, the cultural dynamics, suggesting older adults might rate their SDM experiences based on feelings of trust and gratitude [58–60], might explain this refinement.

In the current study, our qualitative findings introduced a new concept: how cultural norms shape clinical decision-making in cervical cancer. Because they might induce a cultural tendency towards paternalism in medical practice, where physician authority gets valued over patient autonomy [57]. Thus, ethical commitment and transparency of the health care provider [61] might be misinterpreted as professional inadequacy. Thus, according to our qualitative findings, these cultural domains might constrain the practice of SDM in clinical care provision. Additionally, the qualitative findings have also introduced how emotional stability functions as SDM enabler. The current study did not show a significant statistical association between time (months) since diagnosis and perceived level of SDM, unlike other studies [62–64]. Nevertheless, existing literature reads that prolonged time since diagnosis contributes to a reduction in emotional stability, making it more difficult for patients to participate in their clinical care [65,66]. This aligns with a couple of studies that confirmed that emotional regulation of patients enhances their capacity to participate in SDM, while anxiety and distress diminish active involvement and comprehension [51,54].

Quantitatively, in the current study, women from rural areas had statistically significant lower engagement in their clinical care. This hypothesis has been confirmed by our qualitative findings as healthcare providers witness urban women come with questions that have been raised from various sources of information. Similarly, a study has shown that urban residency is associated with improved health literacy, greater exposure to media, and better access to health care institutions [67], which might explain this convergence. Additionally, studies revealed disparities between urban and rural cancer patients as shown through differences in access to the art of cancer prevention, diagnosis, and treatment [68,69], which might further contribute to the disparities of the SDM practice between the two groups of the population.

Unlike [8] and findings from our thematic analysis, the health literacy of the participants had a negative linear association with the perceived level of SDM. This contradiction emphasizes the differences between qualitative and quantitative methods. Quantitative studies, using standardized tools, often overlook cultural and contextual realities, while qualitative interviews explore deeper thoughts, emotions, and relationships that quantitative scales cannot capture [70], leading to contrasting results. Theoretically, patients with low levels of literacy may also have low skills in participating in SDM unless the provider has good communication skills and the ability to simplify information using visual aids or using the local language [71]. On the other hand, the urgent, protocol-driven nature of cervical cancer treatment [72] may lead patients to misinterpret brief treatment guidance as SDM, thereby assuming high participation in their clinical care.

On the contrary, information overload and avoidant behavior of literate individuals may prevent them from participating in SDM. Further, individuals with a high level of literacy may deem themselves to have enough information, which may make them avoid participating in SDM [73]. Consequently, our findings from the qualitative and quantitative approaches showed that health literacy may not be a linear determinant of SDM but a mediator influenced by communication quality, cultural norms, and patient-provider relationships.

Finally, in the current study, the mean score of trust in oncologists had a significant positive linear association with perceived levels of SDM, which was confirmed by our thematic analysis and a study from Germany among oncology patients [74]. Trust is a fundamental component in clinical care, enabling collaboration and mutual understanding [75,76], making it crucial in the patient-doctor relationship. In our current study, the qualitative findings revealed how dismissive behavior of healthcare providers undermined patients’ trust in their oncologist. However, previous studies have shown that physician empathy and attentiveness have enhanced SDM practice in clinical care [65,77]. Furthermore, in clinical care of cervical cancer, where treatment decisions can be complex and emotionally challenging, trusting the oncologist may be so crucial for the patient. Thus, relational trust may increase the perceived level of SDM despite the lower cognitive and awareness involved.

### Strength and limitations of the study

As for the strength, the current study employed a convergent parallel mixed study, which enables us to understand predicting factors of shared decision-making more. Further, this study design can solve limitations of statistical analysis, as it tends to explore reasons for the predictors. Further, the findings from the study can be validated, as the convergent design used multiple methods, enhancing the credibility of the data.

The study employed a cross-sectional design, limiting its ability to track changes in SDM over time. While a high response rate enhances internal validity, consecutive sampling may introduce selection bias by favoring patients at the clinic and excluding those with limited access or different health-seeking behaviors, affecting generalizability. Conducted at a single tertiary care center, the findings may not represent broader populations, as such centers often handle complex cases unlike primary or rural facilities, where SDM and health literacy may vary. Therefore, replication across multiple sites is necessary to improve external validity and capture context-specific differences. Finally, this exploratory analysis aimed to identify predictors of SDM among women with cervical cancer, and results should be interpreted cautiously to guide future hypothesis-driven research.

## Conclusions and recommendations

In conclusion, this study highlights the understandings of how SDM is practiced in the clinical care of cervical cancer. The integration of the quantitative and the qualitative results revealed that SDM is shaped by communication-related barriers, systemic constraints, and socioeconomic status. Furthermore, family involvement in the care process and cultural norms often diminish patients’ participation in their care process. Similarly, our findings have shown that rural residence further restricts engagement in the clinical care process among women with cervical cancer. Although health status (which was defined by the ECOG score of the patient) and age did not show statistical associations, the qualitative results have shed light on their practical influence on comprehension and involvement in the clinical care process. Trust and emotional wellbeing also emerged as enablers of SDM in the current study, emphasizing the relational dimension of patient–doctor interactions.

Therefore, to improve the practice of SDM in clinical care of cervical cancer, interventions should focus on strengthening physician communication skills, cultural sensitivity of clinical approach, and balancing family involvement and the concept of patient autonomy. Furthermore, systematic interventions should focus on enhancing privacy in clinical care of cervical cancer and ensuring continuity of care in teaching hospitals like TASH. Targeted interventions on communication skills would also be recommended.

## Abbreviations

IDIs: In-depth Interviews
KIIs: Key-Informant Interviews
LMICs: Low and Middle Income Countries
OPD: Out Patient Department
QOL: Quality of Life
REC-EB-SPH: Research Ethics Committee at Department of Epidemiology and Biostatics, School of Public Health
SDM: Shared decision Making
SSA: Sub-Sharan Africa
TASH: Tikur Anbessa Specialized Hospital.
